# Selection of Agar Reagents for Medium Solidification Is a Critical Factor for Metal(loid) Sensitivity and Ionomic Profiles of *Arabidopsis thaliana*

**DOI:** 10.3389/fpls.2020.00503

**Published:** 2020-05-15

**Authors:** Shimpei Uraguchi, Yuka Ohshiro, Yuto Otsuka, Hikari Tsukioka, Nene Yoneyama, Haruka Sato, Momoko Hirakawa, Ryosuke Nakamura, Yasukazu Takanezawa, Masako Kiyono

**Affiliations:** Department of Public Health, School of Pharmacy, Kitasato University, Tokyo, Japan

**Keywords:** agar reagent, Arabidopsis, *cad1-3*, ionome, metal sensitivity, metal toxicity

## Abstract

For researchers in the plant metal field, the agar reagent used for the solid plate medium is a problematic factor because application of different agar types and even a different lot of the same agar type can mask the plant metal-related phenotypes and impair the reproducibility. In this study, we systematically assessed effects of different agar reagents on metal(loid) sensitivity and element accumulation of the Arabidopsis metal sensitive mutants. Three established mutants (*cad1-3*, *cad1-6*, and *abcc1/2*), and three different types of purified agar reagents (Type A, Type E, and Nacalai) with two independent batches for each reagent were subjected to the analyses. First, we found that element concentrations in the agar reagents largely varied among the agar types. Then the effects of agar reagents on the mutant metal(loid)-sensitivity were examined under As(III), Hg(II), Cd(II), and excess Zn(II) conditions. A significant variation of the mutant metal(loid)-sensitivity was observed among the different agar plates but the variation depended on the combination of metal(loid) stress and agar reagents. Briefly, the type-dependent variation was more evident under As(III) and Hg(II) than Cd(II) or excess Zn(II) conditions. A lot-dependent variation was also observed for Type A and Type E but not for Nacalai: hypersensitive phenotypes of *cad1-3*, *cad1-6*, and *abcc1/2* under As(III) or Hg(II) treatments were diminished when different batches of the Type A or Type E agar types were used. We also found a significant variation of As and Hg accumulation in the wild-type and *cad1-3*. Plant As and Hg concentrations were remarkably higher and the difference between the genotypes was more evident when grown with Type A agar plates. We finally analyzed ionomic profiles in the plants exposed to As(III) stress. Agar-type specific ionomic changes in *cad1-3* were more observed with the Type A plates than with the Nacalai plates. The presented results overall suggest that suitability of agar reagents for metal(loid)-related phenotyping depends on the experimental design, and an inappropriate selection of agar reagents can mask even very clear phenotypes of the established mutant like *cad1-3*. We also discuss perspectives on the agar problem in the plant metal study.

## Introduction

Contamination of environments or foods by heavy metals and metalloids such as arsenic (As), cadmium (Cd), mercury (Hg), lead (Pb), and zinc (Zn) is a worldwide concern as environmental, agricultural or health risks (Järup, 2003; Clemens, 2019). As health risks, As, Cd, Hg, and Pb rank in the top 10 on the US Agency for Toxic Substances and Disease Registry (ATSDR) 2017 Priority List of Hazardous Substances.^1^ Plant-derived foods (mainly cereal grains) are often major sources of the toxic elements like As and Cd among the general people (Tsukahara et al., 2003; Gilbert-Diamond et al., 2011). As for Hg, the methylated form is a well-known toxicant mainly accumulated in seafoods, but its inorganic form [Hg(II)] is also toxic and substantially detected from cereal grains and vegetables harvested from the contaminated environments (Li et al., 2017; Tang et al., 2018). Pb is also a toxic metal but its accumulation in plants hardly becomes a problem unlike the other elements. It is firstly because phytoavailability of Pb in soils is much lower compared to those of As, Cd, Hg, and Zn (of the inorganic forms) (Xian, 1987; Jing et al., 2008; Stroud et al., 2011) and secondary, probably due to the very low uptake and within-plant mobility of Pb (Kumar and Prasad, 2018). Zn is an essential element for both plant and human but its excess causes toxicity: excess Zn treatment leads to over-accumulation of Zn and inhibits plant growth by disrupting mineral homeostasis and vacuolar functions (Fukao and Ferjani, 2011; Fukao et al., 2011). With these reasons, the plant studies of toxic metal(loid)s rather focus on As, Cd, Hg, and excess Zn than Pb.

Molecular understanding of plant metal transport and tolerance is an important basis to control plant metal responses for further mitigating the various toxic element-related risks (Clemens, 2019). Genetic analyses of the model plants Arabidopsis and rice largely contribute to identifying molecules playing crucial roles in plant metal transport and tolerance (see reviews by Krämer et al., 2007; Uraguchi and Fujiwara, 2013; Ma et al., 2014; Clemens and Ma, 2016; Bashir et al., 2016; Yamaji and Ma, 2017). Phenotyping loss-of-function mutants of candidate player genes is the key to such genetic analyses elucidating the molecular functions *in planta*. Under the laboratory conditions, for both forward and reverse genetic experiments of Arabidopsis, in particular, artificial solid plate medium is a useful assay system to observe mutant plant responses against metal(loid) stress. Murashige-Skoog (MS) based solid medium is widely used for general plant experiments, however, for phenotyping assay under metal(loid) stress, diluted media with lower ionic strength are often preferred (Hou et al., 2005; Tazib et al., 2009; Huang et al., 2010; Yamaguchi et al., 2016). Medium dilution reduces the competitive interaction of the supplemented ions in the medium and sharpens the toxic effects of the target metal(loid). Among one-tenth strength modified Hoagland medium is one of the promising media for various metal(loid)-related phenotyping (Tennstedt et al., 2009; Fischer et al., 2014; Kühnlenz et al., 2014; Uraguchi et al., 2018, 2019a,b). The assay with this medium highlights the metal-sensitive phenotypes of several mutants, which are not visible under the MS-based conditions (Tennstedt et al., 2009; Fischer et al., 2014).

In addition to the medium composition, the selection of agar reagents for plate solidification is another medium-related factor affecting plant responses to mineral/metal stress. This point has been examined by a few previous studies in regard to nutrient deficiency responses of Arabidopsis: elemental composition of agar reagents largely varies among types or batches of agar reagents, which consequently affects plant responses and phenotypes to macro- and micro-nutrient starvation (Jain et al., 2009; Gruber et al., 2013). As for heavy metal toxicity assay, it would be also vaguely recognized among researchers in the plant-metal community that types and lots of agar reagents can influence metal-related phenotypes and impair the reproducibility of experiments. Indeed, we have occasionally experienced agar type- and lot-dependent variation of metal-sensitive phenotypes of Arabidopsis.

In this study, using a set of Arabidopsis metal-sensitive mutants, we systematically assessed variation of metal(loid)-related phenotypes attributed to different agar types and batches. The further aim of the study was to promoting awareness to the plant-metal community about the importance of agar reagents used for metal-related phenotyping. For this purpose, the established metal-sensitive Arabidopsis mutants were subjected to the assay: *cad1-3* and *cad1-6* are loss-of-function alleles of AtPCS1, the Arabidopsis major phytochelatin (PC) synthase (PCS). The two alleles respond differently to As(III), Cd(II) and excess Zn(II) stress (Tennstedt et al., 2009; Uraguchi et al., 2018). *abcc1*/*abcc2* (hereafter *abcc1/2*) is the double knock-out mutant of AtABCC1 and AtABCC2 that function as PC-metal(loid) complex transporters on tonoplast (Song et al., 2010; Park et al., 2012). We tested three types of agar reagents often used for metal-related phenotyping as well as two different lots for each type. A variation of plant growth under As(III), Hg(II), Cd(II), and excess Zn(II) attributed to agar reagents, and elemental profiles of plants and agar reagents were analyzed.

## Materials and Methods

### Agar Reagents and Ionomic Profiles

Three types of agar reagents and two independent lots for each type were subjected to the study: agar Type A (Sigma-Aldrich, catalog no. A4550-500G, lot nos. SLBL4283V and SLBJ9623V, hereafter Type A-1 and Type A-2, respectively), agar Type E (Sigma-Aldrich, catalog no. A4675-500G, lot nos. SLBN1798V and SLBL0898V, hereafter Type E-1 and Type E-2, respectively) and purified agar (Nacalai Tesque, catalog no. 01162-15, lot nos. M7B5170 and M7K8912, hereafter Nacalai-1 and Nacalai-2, respectively). These agar reagents are often used for analyzing Arabidopsis responses to metal toxicity or mineral stress.

To obtain ionomic profiles of the agar reagents, elemental concentrations of the agar reagents (Type A-1, Type A-2, Type E-1, Type E-2, Nacalai-1, and Nacalai-2) were determined as described previously (Uraguchi et al., 2018, 2019a). 100 mg of each agar reagent was wet-digested with 3ml of HNO_3_. After filling up to 5 ml with 2% HNO_3_, total Hg concentrations in the digested samples were quantified by a cold vapor atomic absorption spectrometer (CV-AAS, HG-400, Hiranuma). Concentrations of elements other than Hg were quantified by an inductively coupled plasma-optical emission spectroscopy (ICP-OES, iCAP7400Duo, Thermo Fisher Scientific).

### Plant Materials and Growth Conditions

*Arabidopsis thaliana* wild type (Col-0), the *AtPCS1* null mutant *cad1-3* (Howden et al., 1995), the *AtPCS1* T-DNA insertion line *cad1-6* (Tennstedt et al., 2009) and the *AtABCC1* and *AtABCC2* double knockout mutant line *abcc1/2* (Song et al., 2010) were used for the growth assay. For elemental analyses, Col-0 and *cad1-3* were used.

One-tenth strength modified Hoagland medium was used for plant cultivation [100 μM (NH_4_)_2_HPO_4_, 200 μM MgSO_4_, 280 μM Ca(NO_3_)_2_, 600 μM KNO_3_, 5 μM Fe- N, N′-di-(2-hydroxybenzoyl)-ethylenediamine-N,N′-diacetic acid (HBED), 1% (w/v) sucrose, 5 mM MES, pH 5.7] (Tennstedt et al., 2009; Kühnlenz et al., 2014). For metal(loid) sensitivity assay, essential microelements other than Fe were not added to the medium to avoid possible interaction between the metal(loid) added. To prepare the plants for elemental analyses, the following essential microelements were additionally supplemented to the medium (4.63 μM H_3_BO_3_, 32 nM CuSO_4_, 915 nM MnCl_2_, 77 nM ZnSO_4_, 11 nM MoO_3_). The concentration of agar reagents used for medium solidification was 1% (w/v) for Type A and Type E, and 1.5% (w/v) for Nacalai. The higher concentration of the Nacalai agar was determined to obtain sufficient gel strength to reduce root penetration into the medium. Plastic square plates (AW2000, Eiken Chemical) were used for the agar plate preparation.

Arabidopsis seeds were surface sterilized by 5 min-immersion with 70% (v/v) ethanol, followed by 1-min immersion with 99.5% ethanol (Sotta and Fujiwara, 2017). After air-drying, the sterilized seeds resuspended with 0.1% (w/v) agar were sown on agar plates (seven seeds of each genotype per plate for sensitivity assay, and 36 seeds of a single genotype per plate for ionome assay). After 2 days stratification at 4°C, plants were grown vertically in a growth chamber (16 h light/8 h dark, 22°C) as described elsewhere.

### Metal(loid) Sensitivity Assay

To examine the effects of agar reagents on metal(loid) sensitivity of the Arabidopsis metal-hypersensitive mutants, the growth assay was conducted by modifying the method previously described for As and Cd treatment (Uraguchi et al., 2017, 2018). For this assay, Col-0, *cad1-3*, *cad1-6*, and *abcc1/2* were tested with all six agar reagents (Type A, Type E and Nacalai with two lots, respectively). Plants were grown for 12 days on agar plates containing different concentrations of As(III) as NaAsO_2_ (1 and 1.5 μM), Hg(II) as HgCl_2_ (10 and 15 μM), Zn (II) as ZnSO_4_ (40 and 50 μM) or Cd(II) as CdCl_2_ (2 and 3 μM). These metal(loid) concentrations were determined based on the previous reports (Tennstedt et al., 2009; Uraguchi et al., 2017, 2018, 2019b) as well as our preliminary experiments. The selected treatments were expected to maximize the phenotypic differences between the wild-type and the mutants (thus not lethal to the wild-type Col-0 during the experimental period). Two different levels for each metal(loid) were examined to cover possible variation of the plant growth derived from the “agar-effects.” The plates without the addition of the metal(loid) served as control. After 12 days cultivation, plants were photographed, and plant growth was assessed by primary root length and seedling (roots and shoots) fresh weight measurements. For the respective genotypes, root length was measured for each plant, and seedling fresh weight was measured for each plate.

### Elemental Accumulation in Plants

To examine the effects of agar reagents on plant elemental accumulation, elemental concentrations in the plants were compared among Type A-1, Type A-2, Nacalai-1, and Nacalai-2. The plant elemental analysis was conducted as described previously (Uraguchi et al., 2018, 2019a) with a slight modification. Col-0 and *cad1-3* were grown on the control plates for 10 days. Uniformly grown seedlings were then transferred to control plates or plates containing 5 μM As (III) or 10 μM Hg(II) and grown for additional 4 days. At harvest, roots and shoots from each plate (15 seedlings per genotype for control and Hg(II) treatment, and 30 seedlings per genotype for As(III) treatment) were separately pooled as a single sample. Shoot samples were washed with MilliQ water twice. Root samples were subjected to sequential washing procedures: roots were desorbed for 10 min each in ice-cold MilliQ water, 20 mM CaCl_2_ (twice), 10 mM EDTA (pH 5.7) and MilliQ water. Harvested roots and shoots were dried at 50°C before acid digestion at least for a few days. Dried plant samples (3–10 mg for control and Hg(II) treatment samples, and 5–20 mg for As(III) treatment samples) were wet-digested with 3ml of HNO_3_. After filling up to 5 ml with 2% HNO_3_ in a plastic tube, total Hg concentrations in the digested samples obtained from the Hg(II) treatment were quantified by CV-AAS. For the As(III) treated plant samples, multiple elemental concentrations including As were quantified by ICP-OES.

### Statistical Analyses

The R software (ver. 3.5.1) was used for statistical analyses. The root growth and seedling fresh weight data obtained from the growth assay was analyzed by one-way ANOVA, followed by Tukey’s HSD (*p* < 0.05) to examine the significance of differences among the plant genotypes grown under respective metal(loid) stress and each agar reagent. The Hg and As concentrations in the plant were also analyzed by one-way ANOVA, followed by Tukey’s HSD (*p* < 0.05) to examine the effects of the agar reagents and the plant genotypes. The plant ionomic data were analyzed by three-way ANOVA (*p* < 0.05) to verify the effects of genotypes, As(III) treatment and agar reagents, and the interaction of these factors. A *post-hoc* analysis by Tukey’s HSD (*p* < 0.05) was conducted for each agar reagent to examine the *cad1-3* specific ionomic changes under As(III) stress.

## Results

### Ionomic Profiles of the Agar Reagents

**FIGURE 1:**
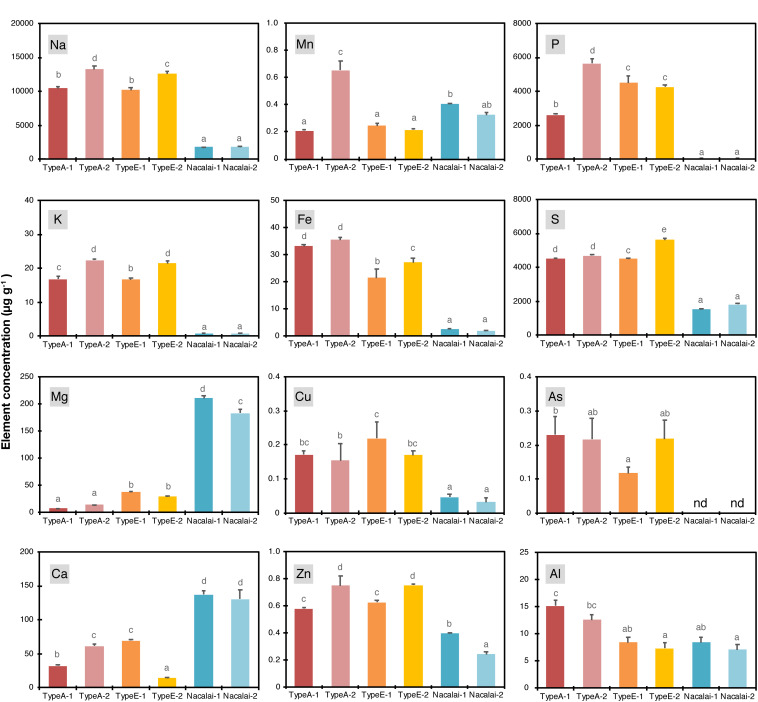
Concentrations of Na, K, Mg, Ca, Mn, Fe, Cu, Zn, P, S, As, and Al in the agar regents (μg g^–1^). Data represent means with standard deviation of at least three independent measurements. Means sharing the same letter are not significantly different within each panel (*P* < 0.05, Tukey’s HSD). n.d., not detected.

We expected a variation of ionomic profiles in the agar reagents used in this study, as previously reported for other agar reagents (Jain et al., 2009; Gruber et al., 2013). We first quantified total element concentrations in the agar reagents (Figure 1). The measured elements were briefly classified into two groups: the first group included sodium (Na), potassium (K), Fe, copper (Cu), Zn, phosphorus (P), sulfur (S), and As. These element concentrations were higher in Type A and Type E agars than in Nacalai agars. This tendency was drastic for Na, K, Fe, and P (up to 7-, 27-, 20-, and 100-fold higher in Type A or Type E than Nacalai, respectively). The second group included magnesium (Mg) and calcium (Ca), which concentrations were drastically higher in Nacalai agars. A pattern of manganese (Mn) was different from these two groups. Nacalai agars contained relatively higher Mn concentrations than Type A-1, and Type E agars but Type A-2 showed the highest concentration. Aluminum (Al) concentrations were almost 2-fold higher in Type A agars than the other agars.

We then calculated molar concentrations of the respective elements in the one-tenth strength modified Hoagland medium supplemented with all micronutrients (Supplementary Figure S1). The agar concentration used for the calculation was 1% (w/v) for Type A and Type E, and 1.5% (w/v) for Nacalai agars, which were the same concentrations used for the plate preparation in this study. This calculation did not take account of chemical forms and phytoavailability of the agar-derived elements, thus it evaluated maximum additive elemental load to the medium derived from the agar reagents. Proportions of the additive load to the basal medium were remarkably higher in Fe, Cu, Zn, P, and S. The agar-derived loads of Fe, Cu, and Zn were almost equal to the basal medium levels in the highest cases. The addition of Type A and Type E drastically increased total P and S concentrations in the medium (up to 20- and 10-fold increase, respectively). The increase of Fe, Cu, P, and S by Nacalai agar addition was much more moderate compared to the case of Type A and Type E agars. In contrast, the additive levels of Mg and Ca were estimated to be up to 65 and 18% of the respective basal concentrations in the medium for Nacalai agars but were much less for Type A and Type E agar reagents.

For non-essential elements, Na and As showed a similar pattern: higher contents in Type A and Type E and lower in Nacalai agars (Figure 1). But their molar concentrations in the medium were < 6 mM for Na and < 0.03 μM for As (Supplementary Figure S1). These values were way below the level to cause toxicity to plants. Al concentrations were ~2-fold higher in Type A agars compared to Type E and Nacalai agar reagents (Figure 1). But molar concentrations of Al in the medium were in a range of 3–5 μM (Supplementary Figure S1), which was again below the toxic levels used in many experiments. In addition to the elements presented in Figure 1 and Supplementary Figure S1, Cd, Hg, and Pb were also quantified but concentrations of these elements in the agar reagents were below the quantification limits of our ICP-OES (for Cd and Pb) and CV-AAS (for Hg) systems.

A lot-dependent variation of the elemental profiles was also found but not so drastic as the type-to-type variation mentioned above. A variation between Nacalai agars was overall very minor. In contrast, compared to Type A-1, Type A-2 contained higher levels of Na, K, Mg, Ca, Mn, Fe, Zn, and P (Figure 1), but the difference in the medium concentration basis was little except for P (Supplementary Figure S1). The total P concentration in the medium was 2-fold higher with Type A-2 than Type A-1 (Supplementary Figure S1). Type E-2 contained higher levels of Na, K, Fe, Zn, S, and As and lower levels of Mg and Ca than Type E-1 (Figure 1). However, comparing the concentrations in the medium, the difference between the Type E batches was not that much evident (Supplementary Figure S1).

### Agar-Dependent Variation of As(III)-Sensitive Phenotypes

**FIGURE 2:**
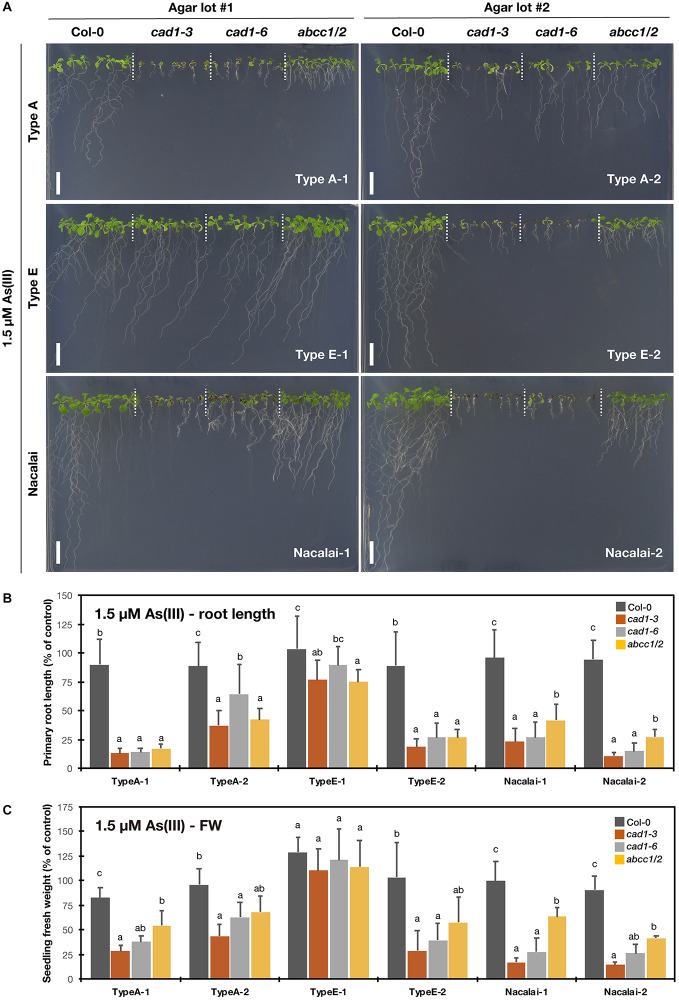
As(III)-sensitivity of the phytochelatin-related Arabidopsis mutants on the agar plates prepared with different agar reagents. Three types of agar reagents with two independent lots for each type were tested. Col-0, *cad1-3*, *cad1-6* and *abcc1/2* were grown on the agar medium containing 1.5 μM As(III) for 12 days. **(A)** Phenotypes of Col-0, *cad1-3*, *cad1-6*, and *abcc1/2*. Scale bars = 1 cm. **(B,C)** Relative primary root length **(B)** and fresh weight of seedlings **(C)** of Col-0, *cad1-3*, *cad1-6*, and *abcc1/2*. Values are shown as percentage of each control. Data represent means with SD from at least three independent experiments (*n* = 19 – 28 for root length, and *n* = 3–4 for seedling fresh weight). Means sharing the same letter are not significantly different within each agar reagent (*P* < 0.05, Tukey’s HSD).

We then tested the metal(loid)-sensitive phenotypes of the established mutants (*cad1-3*, *cad1-6*, and *abcc1/2*) with six agar reagents (Type A-1, A-2, Type E-1, E-2, Nacalai-1, and Nacalai-2) and four metal(loid) species [As(III), Hg(II), Zn(II), and Cd(II)]. The plant growth under control conditions was little affected by the different agar reagents (Supplementary Figures S2–S4). On the other hand, an agar-dependent variation of the metal(loid)-sensitivity was prominently observed for As(III). As(III)-sensitivity assay was conducted under 1 μM (Supplementary Figure S5) and 1.5 μM As(III) treatments (Figure 2). We previously demonstrated comparable As(III)-hypersensitivity of *cad1-3*, *cad1-6*, and *abcc1/2* under these As(III) conditions (Uraguchi et al., 2018). The mutants stably exhibited the As(III)-hypersensitive phenotypes when grown on the plates prepared with Nacalai agars (Figure 2A and Supplementary Figure S5A). The primary root length (Figure 2B and Supplementary Figure S5B) and seedling fresh weight (Figure 2C and Supplementary Figure S5C) of the mutants were severely reduced by the As(III) treatments when either Nacalai-1 or Nacalai-2 was used for the plate preparation. Under 1.5 μM As(III) treatment, *abcc1/2* slightly showed intermediate As(III) sensitive phenotypes compared to the AtPCS1 mutants *cad1-3* and *cad1-6* and this trend was visible on the plates with both Nacalai-1 and Nacalai-2 agars.

In contrast, a lot-dependent variation of the As(III)-sensitivity was observed for Type A and Type E agar reagents. The application of Type A-2 for the medium preparation diminished the As(III) sensitivity of the mutants, whereas the phenotypes on the plates with Type A-1 were similar to those obtained from the Nacalai plates (Figure 2 and Supplementary Figure S5). The lot-to-lot variation of Type E agar was more serious. The As(III)-sensitive phenotypes of *cad1-3*, *cad1-6*, and *abcc1/2* were clearly observed with Type E-2 agar plates even under 1μM As(III) treatment, however, the effects of As(III) exposure on the plant growth were barely visible when Type E-1 was used for the medium preparation (Figure 2A and Supplementary Figure S5A). There was no significant difference of the seedling fresh weight between Col-0 and the mutants under both As(III) conditions (Figure 2C and Supplementary Figure S5C) and the difference of the root length among the genotypes was significant but very little (Figure 2B and Supplementary Figure S5B).

### Agar-Dependent Variation of Hg(II)-Sensitive Phenotypes

**FIGURE 3:**
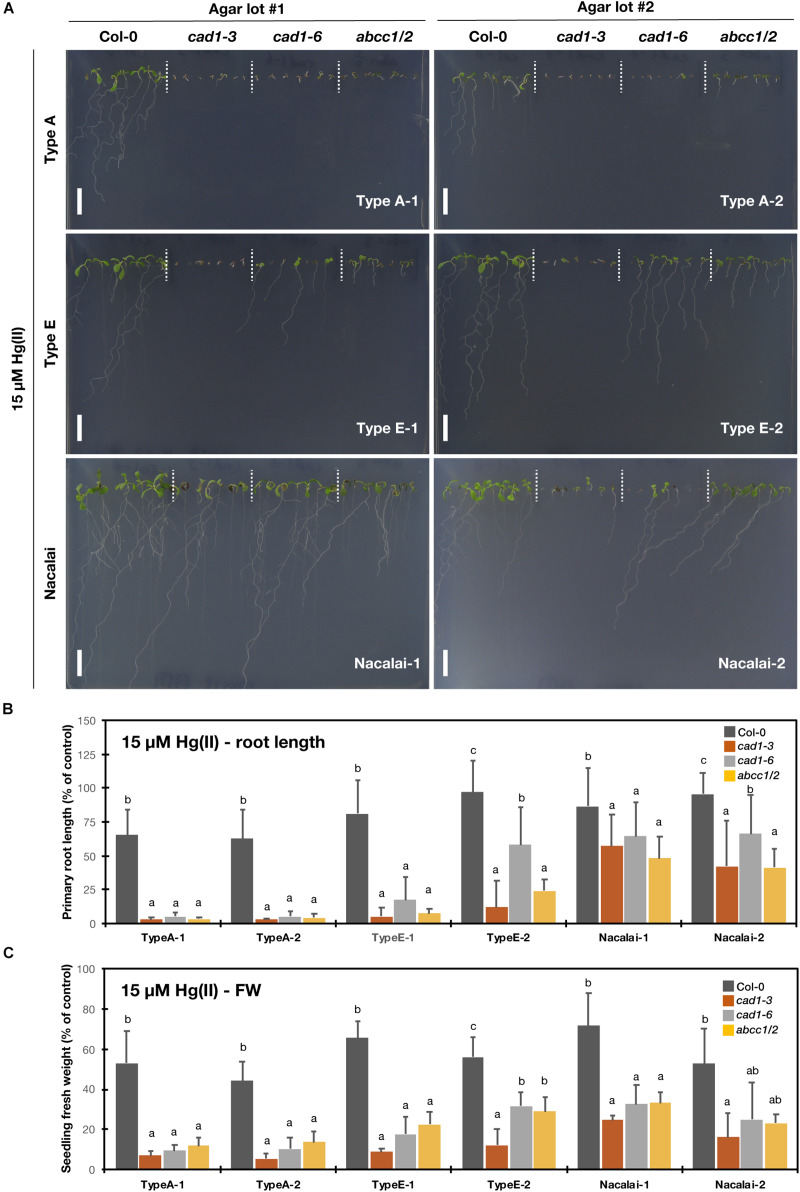
Hg(II)-sensitivity of the phytochelatin-related Arabidopsis mutants on the agar plates prepared with different agar reagents. Three types of agar reagents with two independent lots for each type were tested. Col-0, *cad1-3*, *cad1-6*, and *abcc1/2* were grown on the agar medium containing 15 μM Hg(II) for 12 days. **(A)** Phenotypes of Col-0, *cad1-3*, *cad1-6*, and *abcc1/2*. Scale bars = 1 cm. **(B,C)** Relative primary root length **(B)** and fresh weight of seedlings **(C)** of Col-0, *cad1-3*, *cad1-6*, and *abcc1/2*. Values are shown as percentage of each control. Data represent means with SD from at least three independent experiments (*n* = 13–28 for root length, and *n* = 3–4 for seedling fresh weight). Means sharing the same letter are not significantly different within each agar reagent (*P* < 0.05, Tukey’s HSD).

The plant growth was examined under 10 μM (Supplementary Figure S6) and 15 μM Hg(II) treatments (Figure 3). It has been demonstrated that *cad1-3* and *abcc1/2* are similarly hypersensitive to Hg(II) (Ha et al., 1999; Park et al., 2012). And we found that Hg(II) was the second metal species showing the agar-dependent variation of the mutant hypersensitivity. As a major difference from the case of As(III) treatment, the agar type-dependent variation under Hg(II) stress was more evident than the variation between the batches of the respective agar types. The mutants growth was less inhibited by the Hg(II) treatments on the Nacalai agar plates compared to those of other agar types, whereas the mutant plants grown on the plates with Type A or Type E agars showed severe growth reduction. A variation of the root length among the individuals of each genotype was also larger when the Nacalai agars were used (Figures 3A,B).

A lot-dependent variation was again observed between Type E-1 and E-2 as like the case of As(III), but in the opposite direction: under 15 μM Hg(II) treatment, the growth of *cad1-6* and *abcc1/2* was more severely inhibited on the Type E-1 plates than on the Type E-2 plates (Figure 3). The hypersensitive phenotypes of the tested mutants were remarkably and stably observed on the Type A plates. The root and shoot development of the mutants was nearly completely impaired after germination even under lower Hg(II) treatment and the application of different lots of Type A agar did not change the phenotypes (Figure 3 and Supplementary Figure S6).

### Agar-Dependent Variation of Excess Zn(II)-Sensitive Phenotypes

**FIGURE 4:**
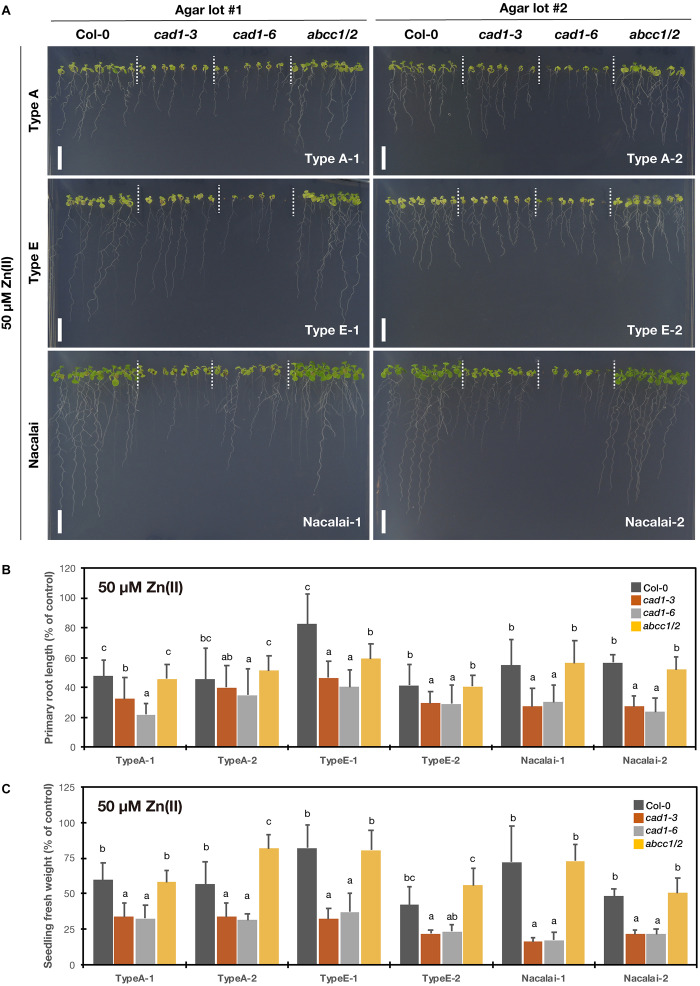
Excess Zn(II)-sensitivity of the phytochelatin-related Arabidopsis mutants on the agar plates prepared with different agar reagents. Three types of agar reagents with two independent lots for each type were tested. Col-0, *cad1-3*, *cad1-6*, and *abcc1/2* were grown on the agar medium containing 50 μM Zn(II) for 12 days. **(A)** Phenotypes of Col-0, *cad1-3*, *cad1-6*, and *abcc1/2*. Scale bars = 1 cm. **(B,C)** Relative primary root length **(B)** and fresh weight of seedlings **(C)** of Col-0, *cad1-3*, *cad1-6*, and *abcc1/2*. Values are shown as percentage of each control. Data represent means with SD from at least three independent experiments (*n* = 22–28 for root length, and *n* = 3–4 for seedling fresh weight). Means sharing the same letter are not significantly different within each agar reagent (*P* < 0.05, Tukey’s HSD).

To test excess Zn(II)-sensitivity of the mutants, the plants were grown under 40 μM (Supplementary Figure S7) and 50 μM Zn(II) treatments (Figure 4). AtPCS1 plays major roles in excess Zn tolerance: *cad1-3* and *cad1-6* exhibit enhanced sensitivity to the excess Zn stress (Tennstedt et al., 2009; Kühnlenz et al., 2016). But phenotypes of *abcc1/2* under excess Zn conditions have not been reported. The root growth and seedling fresh weight of *cad1-3* and *cad1-6* were similarly reduced by 50 μM Zn(II) treatment when the Nacalai agar reagents were used for the medium solidification (Figures 4B,C), as reported previously. Under this condition, the growth of *abcc1/2* was overall similar to that of Col-0. Similar results were obtained for 40 μM Zn(II) treatment (Supplementary Figure S7), suggesting that the sensitivity of *abcc1/2* to excess Zn stress was Col-0 like.

Agar type- and lot-dependent variation was detected for Type A and Type E especially for the root growth: the shorter root length phenotype of *cad1-3* and *cad1-6* under excess Zn(II) was also somewhat observed when grown on the plates prepared with Type A-1 and Type E-1 but less evident on the Type A-2 and Type E-2 plates (Figure 4B and Supplementary Figure S7B). Seedling fresh weight of *cad1-3* and *cad1-6* was also not significantly different from that of Col-0 under 40 μM Zn(II) treatment with Type A-2 and Type E-2 agars (Supplementary Figure S7C).

### Agar-Dependent Variation of Cd(II)-Sensitive Phenotypes

**FIGURE 5:**
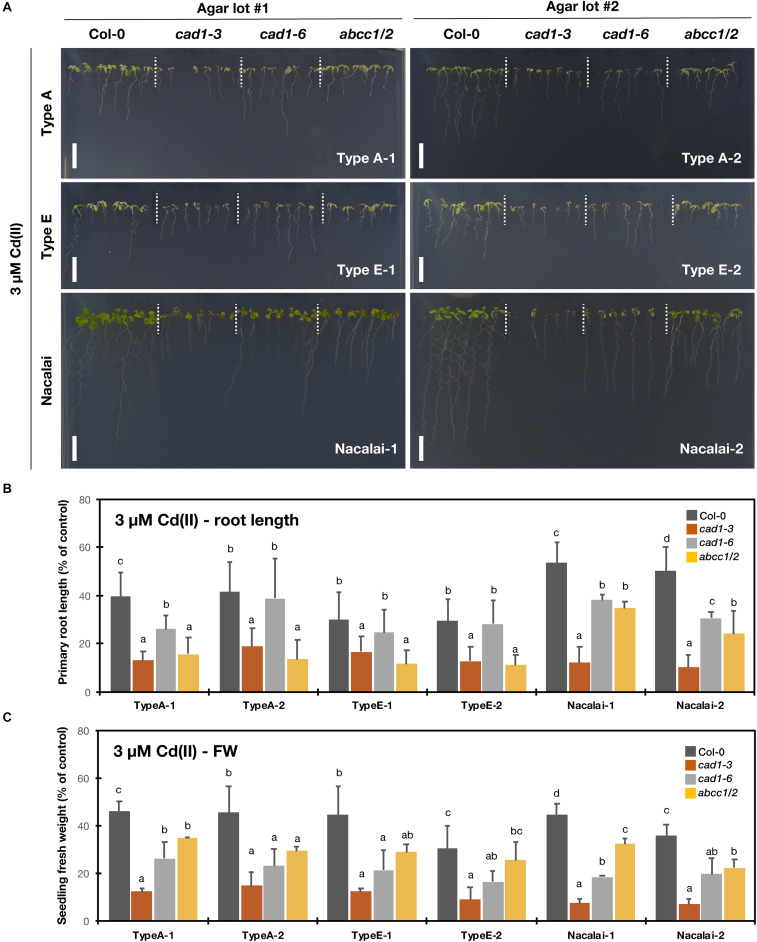
Cd(II)-sensitivity of the phytochelatin-related Arabidopsis mutants on the agar plates prepared with different agar reagents. Three types of agar reagents with two independent lots for each type were tested. Col-0, *cad1-3*, *cad1-6*, and *abcc1/2* were grown on the agar medium containing 3 μM Cd(II) for 12 days. **(A)** Phenotypes of Col-0, *cad1-3*, *cad1-6*, and *abcc1/2*. Scale bars = 1 cm. **(B,C)** Relative primary root length **(B)** and fresh weight of seedlings **(C)** of Col-0, *cad1-3*, *cad1-6*, and *abcc1/2*. Values are shown as percentage of each control. Data represent means with SD from at least three independent experiments (*n* = 24–28 for root length, and *n* = 3–4 for seedling fresh weight). Means sharing the same letter are not significantly different within each agar reagent (*P* < 0.05, Tukey’s HSD).

Compared to the other tested metal(loid)s, the agar-dependent variation of the sensitivity of the mutants under Cd(II) stress was very small. Under both 2 μM (Supplementary Figure S8) and 3 μM Cd(II) treatments (Figure 5), *cad1-3* showed the highest Cd-sensitivity based on the root length and seedling fresh weight quantification irrespective of the agar reagents. *cad1-6* shows a weaker/intermediate Cd-sensitivity compared to *cad1-3* (Tennstedt et al., 2009; Kühnlenz et al., 2016) and indeed, the second allele exhibited the longer root length than *cad1-3* under all the tested agar conditions (Figure 5B and Supplementary Figure S8B). Larger seedling fresh weight of *cad1-6* was significantly observed on the Type A-1, Nacalai-1 and Nacalai-2 plates under both 2 and 3 μM Cd(II), whereas there was no significant difference between *cad1-3* and *cad1-6* when Type A-2, Type E-1, and Type E-2 were used for the medium (Figure 5C and Supplementary Figure S8C).

*abcc1/2* is also a Cd-hypersensitive mutant but the phenotype is reported to be weaker than that of *cad1-3* (Park et al., 2012). In line with this previous result, seedling fresh weight of *abcc1/2* under our assay conditions was generally larger than that of *cad1-3* (Figure 5C and Supplementary Figure S8C). Intermediate root length phenotypes of *abcc1/2* as like *cad1-6* were also observed on the Nacalai plates, however, on the Type A and Type E plates, the *abcc1/2* root length was comparable to that of *cad1-3* (Figure 5B and Supplementary Figure S8B).

### Agar-Dependent Variation of Plant As and Hg Accumulation

Because the growth assay demonstrated considerable agar-dependent variation of the mutant sensitivity especially against As(III) and Hg(II) stress, we hypothesized that the variation was attributed to different accumulation levels of As or Hg in the plants. Thus, we next examined As and Hg accumulation in Col-0 and *cad1-3* grown on the Type A and Nacalai agar plates. *cad1-3* was selected as the representative mutant due to the evident hypersensitive phenotypes under both As(III) and Hg(II) stress. Type E agars were not subjected to this experiment because the remaining Type E-1 was insufficient to complete the experiment. Unlike the sensitivity assay, the established 10-d-old seedlings grown on the control plates were transferred to the plates containing As(III) or Hg(II) to minimize the effects of different sensitivity to As(III) or Hg(II) stress between Col-0 and *cad1-3* on As or Hg uptake and accumulation.

**FIGURE 6:**
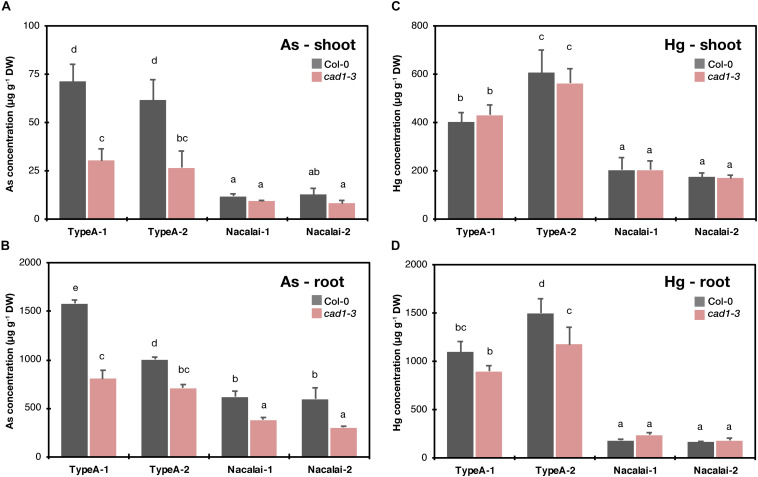
Concentrations of As in shoots **(A)** and roots **(B)** and Hg in shoots **(C)** and roots **(D)** of Col-0 and *cad1-3*. Plants were grown on the control medium prepared with the respective agar reagents for 10 days and then transferred to the medium containing 5 μM As(III) or 10 μM As(III). After 4 days, plants were harvested and As and Hg concentrations in shoots and roots were separately quantified by ICP-OES and CV-AAS, respectively. Data represent means with SD from two independent experiments (*n* = 4). Means sharing the same letter are not significantly different within each panel (*P* < 0.05, Tukey’s HSD).

A variation of As and Hg concentrations in plants was more prominent between agar types than batches (Figure 6). As and Hg concentrations in both shoots (Figures 6A,C) and roots (Figures 6B,D) were generally higher when the Type A agars were used for the medium than the Nacalai agars. Genotypic difference between Col-0 and *cad1-3* was significant for As concentrations in roots irrespective of the agar reagents (Figure 6B) and in shoots harvested from the Type A-1 and A-2 plates (Figure 6A). There was no significant difference between the genotypes for Hg concentrations in shoots and roots, except for that of roots harvested from the Type A-2 plates (Figures 6C,D). In every significant case, *cad1-3* accumulated less As or Hg compared to Col-0.

### Agar-Dependent Variation of Ionomic Profiles of As(III)-Treated Plants

**FIGURE 7:**
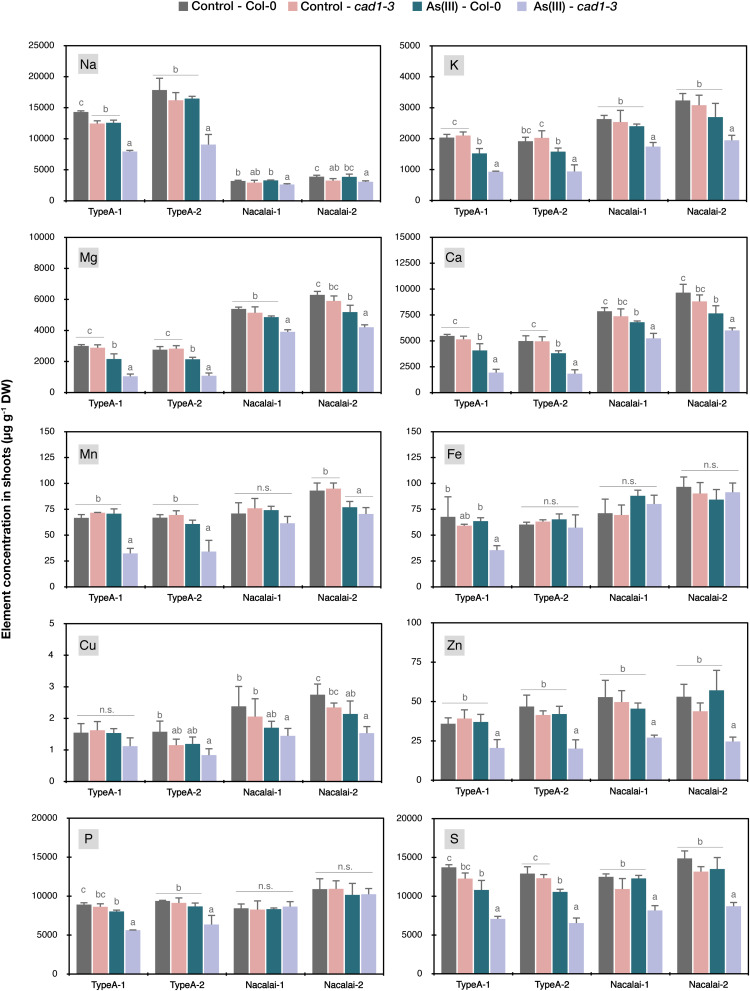
Ionomic profiles in shoots of Col-0 and *cad1-3* under normal conditions and As(III) stressed conditions. Plants were grown on the control medium prepared with the respective agar reagents for 10 days and then transferred to the fresh control medium or the medium containing 5 μM As(III). After 4 days, plants were harvested and concentrations of Na, K, Mg, Ca, Mn, Fe, Cu, Zn, P, and S in shoots were quantified by ICP-OES. Data represent means with SD from two independent experiments (*n* = 4). Means sharing the same letter are not significantly different within each agar reagent (*P* < 0.05, Tukey’s HSD).

Because different As accumulation within the plants seemed not to be a cause for the agar-dependent As(III)-sensitivity variation, we next analyzed the ionomic profiles of the control and As(III)-treated plants grown on the Type A or Nacalai agar plates. Another motivation to conduct ionome analysis of the As(III)-treated plants was our previous result showing that As(III) treatment drastically altered Zn accumulation and distribution specifically in *cad1-3* (Uraguchi et al., 2018). Analyzed elemental concentrations in shoots and roots were shown in Figure 7 and Supplementary Figure S9, respectively, and the obtained ionome data were analyzed by three-way ANOVA as presented in Supplementary Table S1. The effects of As(III) treatment, genotypes (Col-0 and *cad1-3*) and agar reagents (Type A-1, A-2, Nacalai-1, and Nacalai-2) were generally significant over the analyzed elements but were more clearly detected in shoots than in roots (Supplementary Table S1). The typical case was Cu concentrations in roots, which was not significantly affected by any factors. The interaction effects among As(III)-treatment, genotypes, and agar reagents were also tested (Supplementary Table S1): significant interaction between As(III)-treatment and genotypes was detected in most of the elements analyzed except for Fe, Cu, and P in shoots and Na, Mn and Cu in roots. In contrast, for Na, Mn, Fe, Cu, P, and S in shoots and Mn, Fe, Zn, and S in roots, interaction effects between As(III)-treatments and agar reagents were significant. Interaction effects between genotypes and agar reagents were strongly indicated for Na (*P* < 0.001), and weakly indicated for Mn, Zn, and P in shoots (*P* < 0.05). These statistical results indicate that As(III)-treatment induces genotype-specific changes in the accumulation of many elements, and selection of agar reagents influences the degree of the As(III)-induced changes of Na, Mn, Fe, Cu, P, and S in shoots and Mn, Fe, Zn, and S in roots.

We then conducted a *post-hoc* analysis to test significant differences attributed to As(III) treatment and genotypes (Figure 7 and Supplementary Figure S9). As suggested by the three-way ANOVA (Supplementary Table S1), the accumulation of many elements in shoots was significantly altered by the As(III) treatment, especially in *cad1-3*. As(III)-induced reduction of shoot K, Mg, Ca, Zn, and S concentrations more largely in *cad1-3*, irrespective of agar reagents (Figure 7). With the Type A agars, As(III)-induced increase of Zn levels in *cad1-3* roots was larger (Supplementary Figure S9) and alteration of S concentrations by As(III) treatment was significant even in Col-0 (Figure 7 and Supplementary Figure S9). In addition, as Type A agar-specific changes, Na, Mn, and P concentrations in shoots and Mn concentration in roots were significantly reduced by As(III) treatment in *cad1-3* (Figure 7 and Supplementary Figure S9). In contrast, a very slight but significant reduction of Cu concentration under As(III) stress was detected as the only Nacalai agar specific pattern (Figure 7). In comparison to the agar type-dependent variation, a lot-dependent variation was minor. As(III)-dependent reduction in *cad1-3* shoot was significantly clear for Fe on the Type A-1 agar plates but not on the Type A-2 plates (Figure 7), and the effect of agar batches was minor for other cases.

## Discussion

### Significance of Agar Reagents for Metal(loid)-Sensitivity Assay

Gelling agents including agar are one of the basic components of the solid medium for plant experiments, and most of the reports do not mention the details of the gelling reagent used for the experiment. However, a large variation of various element contents was previously reported among a number of different gelling reagents, and “carry-on” elemental load derived from gelling reagents is suggested to mask transcriptional and morphological responses of Arabidopsis plants under nutrient-deficient stress (Jain et al., 2009; Gruber et al., 2013). These studies suggest that the selection of proper gelling agents for each nutrient element is critical for analyzing plant responses to nutrient deficiency. Another suggestion by Jain et al. (2009) is switching a cultivation method from agar-plates to hydroponics. This can solve the problems imposed by elemental contamination from gelling reagents.

For studies of plant metal(loid) transport or toxicity, the importance of selecting appropriate gelling reagents seems to be implicitly recognized for similar reasons. Co-existing ions in the medium (and from the agar reagent) can affect root metal uptake through competitive interactions and therefore modulate levels of metal toxicity to plants. Indeed, several papers mentioned specific types of agar reagents used for analyzing plant metal responses (Thomine et al., 2000; Wong and Cobbett, 2009; Nishida et al., 2011; Weber et al., 2013; Lešková et al., 2017; Uraguchi et al., 2017, 2019b). Hydroponic systems are also used for phenotyping of plant metal responses, which are free from the agar-derived elemental contamination. However, especially for studying Arabidopsis plants, the solid plate system still has some advantages over the hydroponic system. In particular, the plate assay enables phenotyping of many Arabidopsis lines in parallel and analyzing young Arabidopsis seedlings even less than one-week-old. It should be noted that a standard hydroponic protocol of Arabidopsis requires a few weeks preculture to establish seedlings before the treatment. The plate-based metal treatment would also reduce the hazardous risks of handling toxic elements because it is a closed safer system and requires fewer volumes of the media compared to the hydroponic system. Thus optimizing the agar-based metal assay system for Arabidopsis especially in terms of agar reagents is significant.

### Selection of Agar Reagents for Mutant Metal(loid) Sensitivity Assay

Three types of agars with two independent batches for each type were examined in this study. Type A (Nishida et al., 2011; Weber et al., 2013; Uraguchi et al., 2019b) and Type E agars (LeBlanc et al., 2012; Sone et al., 2017; Uraguchi et al., 2017) of Sigma-Aldrich, and purified agar of Nacalai Tesque (Uraguchi et al., 2017, 2018, 2019a,b) are agar reagents previously used for toxic metal treatment of Arabidopsis. It should be noted that this “Type-A” agar is different from other “Type A” agars of Sigma-Aldrich (catalog nos. A1296 and P8169) used in the previous studies (Jain et al., 2009; Gruber et al., 2013). The ionomic analysis of the agar reagents used in this study revealed different elemental backgrounds mainly between the agar types (Figure 1). And the agar-derived additive loads of each element to the medium were considerably higher for Mg and Ca in the Nacalai plates, and for Na, Fe, Cu, P, and S in the Type A and Type E plates (Supplementary Figure S1). With these medium conditions, the three established mutant lines of AtPCS1, AtABCC1 and AtABCC2 were tested as the model of hypersensitive mutants to metal(loid)s: AtPCS1 catalyzes synthesis of PCs, metal(loid)-binding peptides and promotes PC-metal(loid) complex formation in cytosol (Ha et al., 1999; Blum et al., 2010) and AtABCC1 and AtABCC2 coordinately pump the PC-metal(loid) complex into vacuoles (Song et al., 2010; Park et al., 2012).

The comprehensive growth assay demonstrated the significant agar-dependent variation of the mutant phenotypes. Overall, Nacalai agar was suggested as the first choice among the tested three types of agar reagents. Firstly, a lot-dependent variation of the mutant growth was little between the two tested lots (Figures 2–5 and Supplementary Figures S5–S8). We also tested two other lots of the Nacalai agar under some conditions, and we found the similar phenotypes of the mutants regardless of the batches (data not shown). Another important advantage of employing Nacalai agar is that the growth of the wild-type Col-0 and the mutants were more clearly distinguishable than when the other agars were used, except for Hg(II) treatment (Figures 2–5 and Supplementary Figures S5–S8). The representative result was of As(III) treatment. *cad1-3* and *cad1-6* are equally sensitive to As(III) treatment showing shorter roots and accumulation of anthocyanins in leaves, whereas *abcc1/2* exhibits slight but yet better shoot growth without apparent anthocyanin accumulation (Uraguchi et al., 2018). These genotypic differences were stably observed with the Nacalai plates but not with either of the two batches of Type A and Type E agars (Figure 2). For Cd(II) stress, the application of the Nacalai agars enabled stable and clear observation of the intermediate Cd-sensitive phenotypes of *cad1-6* (Figure 5 and Supplementary Figure S8), which was previously reported (Tennstedt et al., 2009; Kühnlenz et al., 2016). The same goes for phenotyping of excess Zn(II) stress. The growth difference between Col-0 and the AtPCS1 mutants (*cad1-3* and *cad1-6*) was smaller than the cases of other metal(loid) stress but the equally sensitive phenotypes of *cad1-3* and *cad1-6* were more sharply and stably detected on the Nacalai plates (Figure 4 and Supplementary Figure S7). Even under such conditions, the growth of *abcc1/2* was overall comparable to that of Col-0, without an apparent sign of Zn-hypersensitivity. AtABCC1 and AtABCC2 are crucial players in metal detoxification by mediating sequestration of the PC-metal(loid) complex into vacuoles (Song et al., 2010; Park et al., 2012). However, our results suggest for the first time that AtABCC1 and AtABCC2 do not significantly contribute to excess Zn detoxification. Another vacuolar Zn sequestration pathway is likely more dominant. AtMTP1, a member of the cation diffusion facilitator (CDF) family is a strong candidate (Kobae et al., 2004; Desbrosses-Fonrouge et al., 2005).

As discussed so far, Nacalai agar was suggested as a versatile gelling agent for the toxic metal(loid) sensitivity assay, however, the exception was Hg(II). The application of the Nacalai agar drastically diminished the Hg(II) sensitivity of the mutant plants (Figure 3 and Supplementary Figure S6). Contrastingly the same Hg(II) treatment with Type A and Type E agars severely inhibited the mutant plant growth, thus these agars rather than Nacalai agar are suitable for Hg(II)-sensitivity phenotyping. For As(III), Cd(II), and Zn(II) stress, we found a considerable variation of the mutant growth between the lots of Type A or Type E agars, however, the hypersensitive phenotypes of the mutants were substantially observed when an appropriate batch was used (Figures 2, 4, 5 and Supplementary Figures S5, S7, S8). Thus, Type A or Type E agar would be the alternative choice of a gelling agent for the growth experiments testing various metal(loid) stress, especially when including Hg(II).

### Agar-Dependent Variation of Plant As and Hg Accumulation and Toxicity

Because we observed the agar type-dependent or lot-dependent variation of the mutant phenotypes mainly under As(III) and Hg(II) treatments, we hypothesized that the agar affected uptake and accumulation of As or Hg in the plants, and different accumulation levels of As or Hg resulted in the different growth sensitivity of the mutant. The results of Hg measurement in Col-0 and *cad1-3* likely explain the agar type-dependent variation of Hg(II)-sensitivity (Figure 3 and Supplementary Figure S6, severe toxicity was observed with the Type A plates but not with the Nacalai plates). Hg concentrations in plants were generally higher when Type A agars were used for the plate preparation than Nacalai agars (>2-fold in shoots, and > 4-fold in roots, Figures 6C,D). The enhanced Hg accumulation with the Type A plates would sharpen the toxic symptoms in the mutants which cannot handle Hg(II) ions in the cells due to the loss-of-function mutations of AtPCS1 or AtABCC1 and AtABCC2 (Ha et al., 1999; Park et al., 2012). A possible cause for the agar-dependent variation of plant Hg accumulation is higher Mg and Ca contents in the Nacalai agar plates (Figure 1 and Supplementary Figure S1). Nacalai agars contained remarkably higher levels of Mg and Ca, which resulted in up to 65 and 20% increase of the medium Mg and Ca concentrations, respectively. It is generally believed that root transport systems for essential cations mediate uptake of non-essential divalent metals including Hg(II). Antagonistic inhibition of Hg(II) uptake was reported by co-existing essential cations including Ca (Esteban et al., 2013; Kis et al., 2017). Taken together, it is very likely that higher concentrations of Mg and/or Ca in the Nacalai plates antagonistically affect Hg uptake by the plants, which consequently diminishes the mutant sensitivity under Hg(II) stress when grown on the Nacalai agar plates.

Unlike the case of Hg(II), different As concentrations in plants are not likely the major factor causing the agar-dependent variation of the mutant As(III)-sensitivity, because the As concentration in the plants and growth sensitivity of the mutant were not correlated between Type A and Nacalai agars, and between Type A-1 and A-2. Plant As concentrations were overall lower when grown with the Nacalai plates than with the Type A plates (Figures 6A,B), although the mutant plants exhibited more severe hypersensitivity to As(III) on the Nacalai plates (Figure 2 and Supplementary Figure S5). The lot-dependent variation of plant As accumulation was not found in *cad1-3* between the Type A plates (Figure 6B), although there was a lot-dependent difference of the As(III)-sensitivity with the Type A plates (Figure 2 and Supplementary Figure S5). Similarly, no correlation between plant As accumulation and sensitivity was reported for *sel1-8*, a loss-of-function mutant of a sulfate transporter gene *SULTR1;2* (Nishida et al., 2016). The study suggests that total As concentrations in plants are not a reliable marker for As(III)-hypersensitivity of *sel1-8*, in which reduced sulfate uptake appears to disrupt GSH-based cellular redox maintenance under As(III) stress (Nishida et al., 2016). The agar-dependent variation of the mutant As(III)-sensitivity observed in this study would be also caused by different physiological conditions of the plants like redox activity. Another possibility is interaction between As(III) toxicity and supplied P levels in the medium as suggested by Lou-Hing et al. (2011). Increased P supply to the medium alleviated As(III) toxicity in rice cultivars, although the mechanism remained elusive. The higher P level in the Type A-2 agar compared to the Type A-1 (Figure 1 and Supplementary Figure S1) could be a reason for the As(III)-sensitivity variation between the Type A lots. However, this hypothesis cannot explain the similar phenotypic variation between the Type E lots, because the P levels of Type E-1 and E-2 were equal (Figure 1 and Supplementary Figure S1). Further molecular understanding of the As(III) toxicity in the plants would provide a clue for the growth condition-dependent variation of the As(III)-sensitivity.

Apart from the metal(loid) sensitivity variation, the agar-dependent variation of the plant As and Hg accumulation that we observed (Figure 6) suggests that the selection of agar reagents is also important for phenotyping metal(loid) accumulation. It should be considered that differences of metal(loid) accumulation between wild-type and mutant plants can be masked by a certain agar reagent as we observed for Type A and Nacalai agars. As far as comparing Type A and Nacalai agars, Type A would be a better selection for assessing plant metal(loid) accumulation because the Type A plates can provide higher plant As and Hg accumulation with clear genotypic differences (Figure 6). Moreover, Type A-specific ionomic alterations were indicated as the As(III) toxicity for Na, Mn, Fe, and P, and for Zn and S, the As(III)-induced changes were more evidently detected with the Type A plates (Figure 7 and Supplementary Figure S9). These results suggest that the Type A medium is overall suitable for analyzing plant element accumulation under metal(loid) stress.

### Perspectives on the Agar Matter of Plant Metal Study

The present study conducted a series of experiments using six different agar reagents and three established metal(loid)-sensitive Arabidopsis mutant plants to examine the effects of agar reagents in the medium on the plant responses against toxic metal(loid) stress. Based on the results presented, we can draw conclusions that (i) a “wrong” agar reagent can easily mask phenotypes (plant growth and metal accumulation) of the well-established Arabidopsis mutants, (ii) suitability of gelling agents for metal(loid) stress assay depends on the target metal(loid), and (iii) elemental profiles of agar reagents would help to predict possible “masking effects” derived from elemental contaminants in the agar regents.

Regarding (i), the agar effect is terrifying as we demonstrated that the growth inhibition of *cad1-3*, one of the most evident metal(loid)-sensitive mutants was completely masked by a certain type of agar reagents. Many of other metal(loid)-sensitive mutants would exhibit more moderate phenotypes compared to *cad1-3*, and then phenotyping with an agar reagent causing even mild masking effects would result in “misphenotyping,” and lead to a wrong conclusion. Similarly, the unsuitable medium composition was reported to cause “misphenotyping” of several mutants under Zn(II) and Pb(II) stress (Tennstedt et al., 2009; Fischer et al., 2014). Related to the second conclusion (ii), what makes the agar problem complicated is that it is almost impossible to find a “complete agar” which can be applied for any metal(loid) treatment. Therefore, one should be careful in the choice of gelling agents for their respective experiments. Conducting the phenotyping assay with at least two independent agar reagents would be a solution to avoid such agar-derived “misphenotyping,” however, it is time-consuming. One alternative suggestion is including *cad1-3* for instance as a positive control to every assay. Suitability of the experimental conditions, including a selection of agar reagents, can be judged based on the *cad1-3* performance. Another possible solution to predict the suitability of agar reagents is an analysis of elemental profiles of the reagent to be used, as stated in the third remark (iii). Our data imply that less contaminated agars are less likely to cause “masking effects,” although it is difficult in some cases to point out which element contamination would be critical. This is likely because such “masking effects” are attributed to chemical and physiological interactions between the elements (Lou-Hing et al., 2011; Lešková et al., 2017). Agarose, which is prepared from agar, can be another candidate for medium solidification, because it generally contains less elemental contaminants than agar reagents (Gruber et al., 2013), due to the removal of agaropectin fractions from agar. However, as reported (Gruber et al., 2013) and as we observed in preliminary experiments, the plant growth was not fine and uniform on agarose medium plates.

Incidentally, our ionomic data of agar reagents demonstrated a large variation of the agar-derived load of essential minerals to the medium, supporting the importance of agar selection for nutrient deficiency experiments (Jain et al., 2009; Gruber et al., 2013). Substantial increase of total element concentrations in the medium was expected for Mg, Ca, Fe, Cu, Zn, P, and S depending on the agar reagents (Supplementary Figure S1). As far as comparing plant ionomic data of control conditions (Figure 7 and Supplementary Figure S9), positive correlations were only detected for Mg and Ca between total element concentrations in the medium and plants. For other elements, concentrations in the plants seemed rather unaffected by the elemental contents in the agar reagents. This would be because total concentrations in the agars do not represent phytoavailable fractions and/or uptake and utilization of these elements by plants are tightly regulated. Nevertheless, our ionomic analysis of the agar reagents indicates that Nacalai agars which have less elemental contamination would be a suitable gelling reagent for nutrient deficiency experiments among the agars tested in this study.

In conclusion, the present study demonstrates with a series of data obtained from several agar reagents that selecting a proper agar reagent is important for metal(loid) stress-related phenotypes and ionome of Arabidopsis. We propose to include a reference mutant such as *cad1-3* to the growth assay to avoid “misphenotyping” caused by agar and other experimental factors. We also propose to describe types and lot numbers of agar reagents in the method section when reporting results of metal-sensitivity growth assay or ionome obtained from agar plate culture.

## Data Availability Statement

All datasets generated for this study are included in the article/Supplementary Material.

## Author Contributions

SU conceived and designed the experiments. MK supervised the study. SU, YOt, HT, NY, HS, and MH conducted the experiments. SU, YOh, RN, YT, and MK analyzed the data. SU and MK prepared the manuscript with contributions from all authors.

## Conflict of Interest

The authors declare that the research was conducted in the absence of any commercial or financial relationships that could be construed as a potential conflict of interest.

## Footnotes

1 https://www.atsdr.cdc.gov/SPL/
