# Supplementary material for: Selection of Agar Reagents for Medium Solidification Is a Critical Factor for Metal(loid) Sensitivity and Ionomic Profiles of Arabidopsis thaliana
Source: Front Plant Sci. 2020 May 15;11:503. doi: 10.3389/fpls.2020.00503 (PMC7243937; doi:10.3389/fpls.2020.00503)
Supplement: Supplementary file 1 [file Data_Sheet_1.pdf]

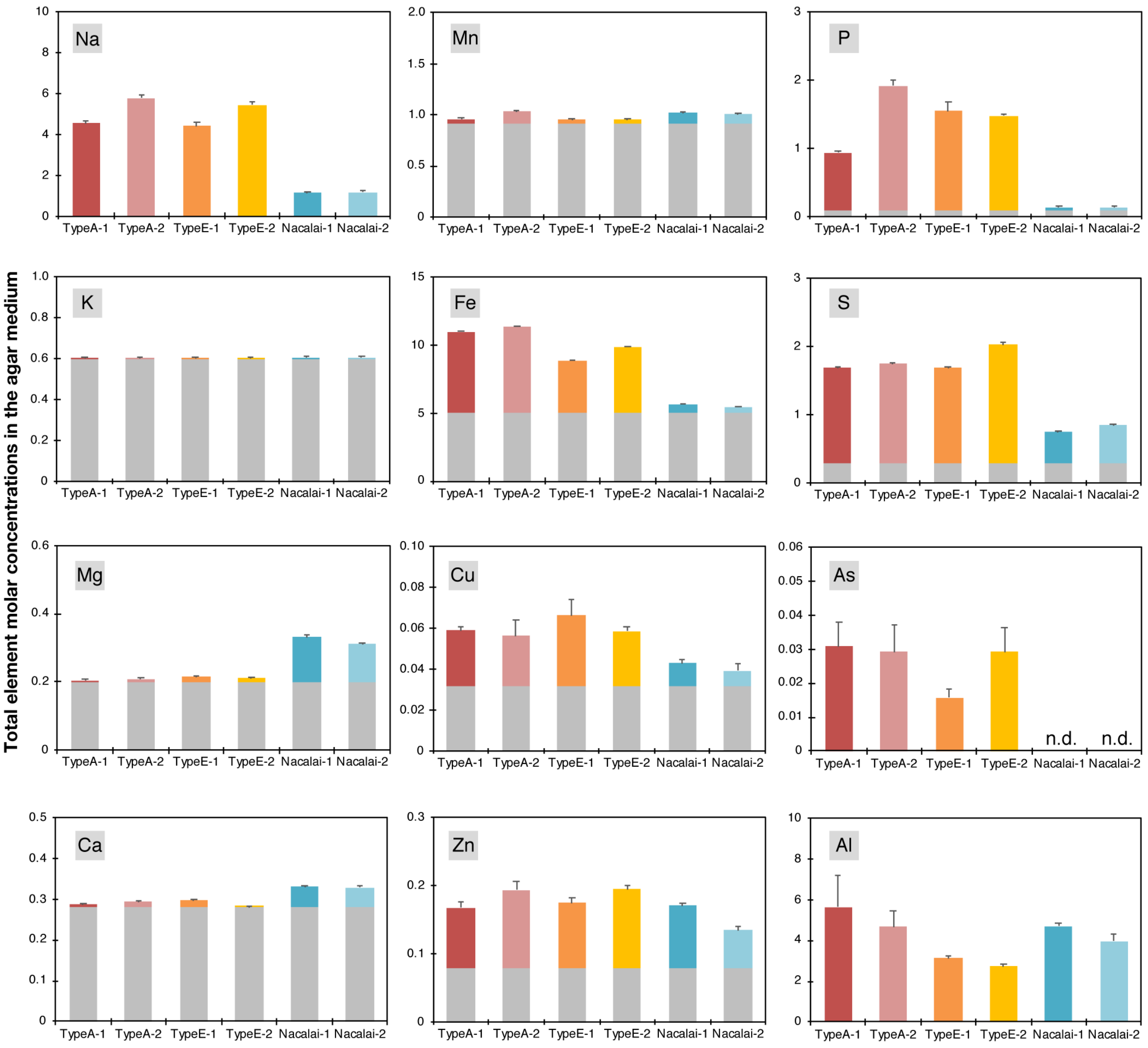

Supplementary Figure S1. Total concentrations of Na, K, Mg, Ca, Mn, Fe, Cu, Zn, P, S, As, and Al in the one-tenth strength modified Hoagland medium supplemented with microelements. Values were calculated based on the elemental concentrations presented in Figure 1, assuming that the concentration of agar reagents used for medium solidification was 1% (w/v) for Type A and Type E, and 1.5% (w/v) for Nacalai. Lower grey bars indicate basal concentrations of each element in the medium and upper colored-bars indicate the additional concentrations derived from the agar reagents. Note that S derived from MES and K from KOH used for pH adjustment were not included to the calculation. Concentrations are presented as mM for Na, K, Mg, Ca, P, and S and µM for Mn, Fe, Cu, Zn, As, and Al, respectively.

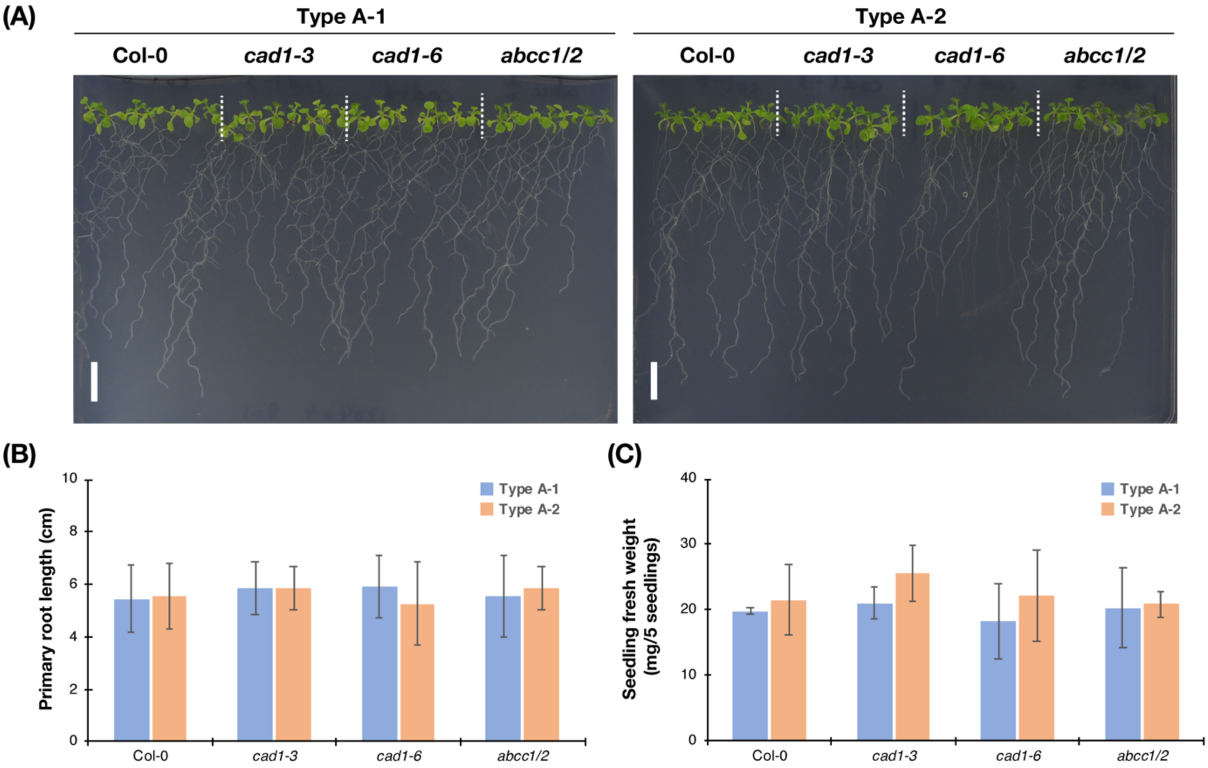

Supplementary Figure S2. Growth of the phytochelatin-related Arabidopsis mutants on the control plates prepared with Type A agars. Col-0, *cad1-3*, *cad1-6* and *abcc1/2* were grown on the agar medium for 12 d. **(A)** Phenotypes of Col-0, *cad1-3*, *cad1-6* and *abcc1/2*. Scale bars = 1 cm. **(B, C)** Primary root length **(B)** and fresh weight of seedlings **(C)** of Col-0, *cad1-3*, *cad1-6* and *abcc1/2*. Data represent means with SD from at least three independent experiments (n = 24 – 28 for root length, and n = 3 – 4 for seedling fresh weight).

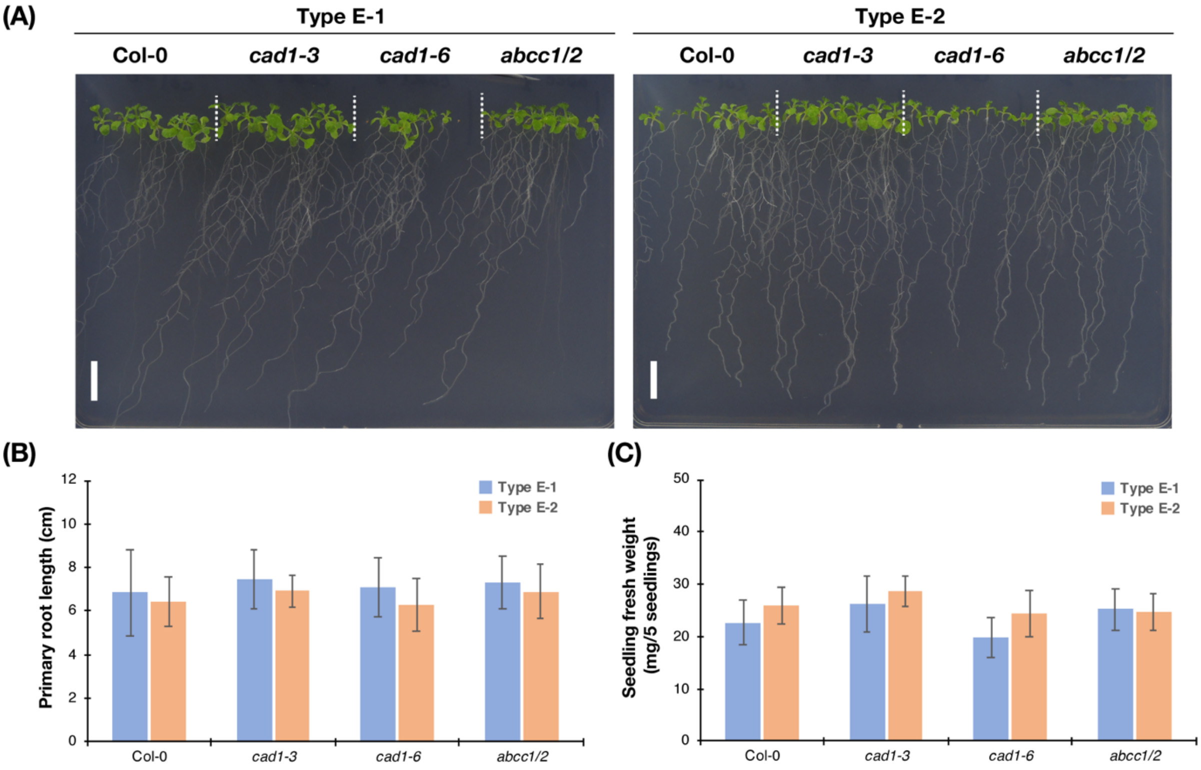

Supplementary Figure S3. Growth of the phytochelatin-related Arabidopsis mutants on the control plates prepared with Type E agars. Col-0, *cad1-3*, *cad1-6* and *abcc1/2* were grown on the agar medium for 12 d. **(A)** Phenotypes of Col-0, *cad1-3*, *cad1-6* and *abcc1/2*. Scale bars = 1 cm. **(B, C)** Primary root length **(B)** and fresh weight of seedlings **(C)** of Col-0, *cad1-3*, *cad1-6* and *abcc1/2*. Data represent means with SD from at least three independent experiments (n = 23 – 28 for root length, and n = 3 – 4 for seedling fresh weight).

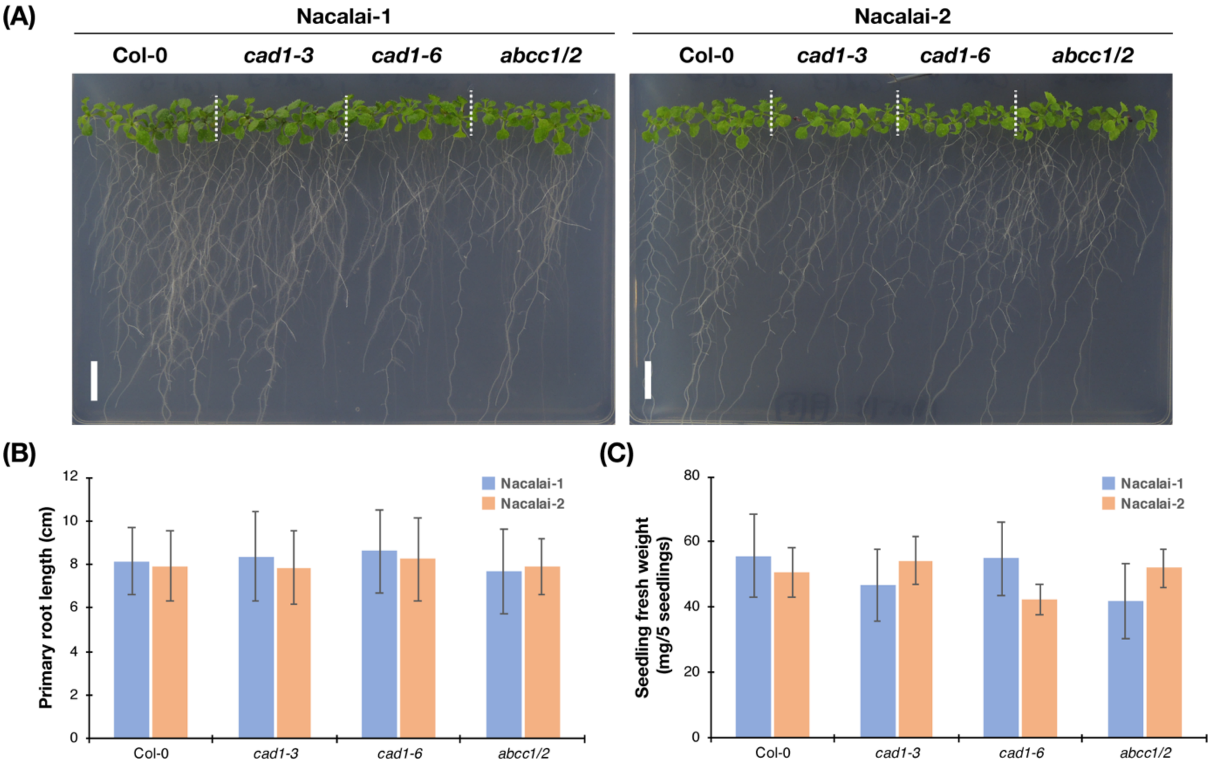

Supplementary Figure S4. Growth of the phytochelatin-related Arabidopsis mutants on the control plates prepared with Nacalai agars. Col-0, *cad1-3*, *cad1-6* and *abcc1/2* were grown on the agar medium for 12 d. **(A)** Phenotypes of Col-0, *cad1-3*, *cad1-6* and *abcc1/2*. Scale bars = 1 cm. **(B, C)** Primary root length **(B)** and fresh weight of seedlings **(C)** of Col-0, *cad1-3*, *cad1-6* and *abcc1/2*. Data represent means with SD from at least three independent experiments (n = 24 – 28 for root length, and n = 3 – 4 for seedling fresh weight).

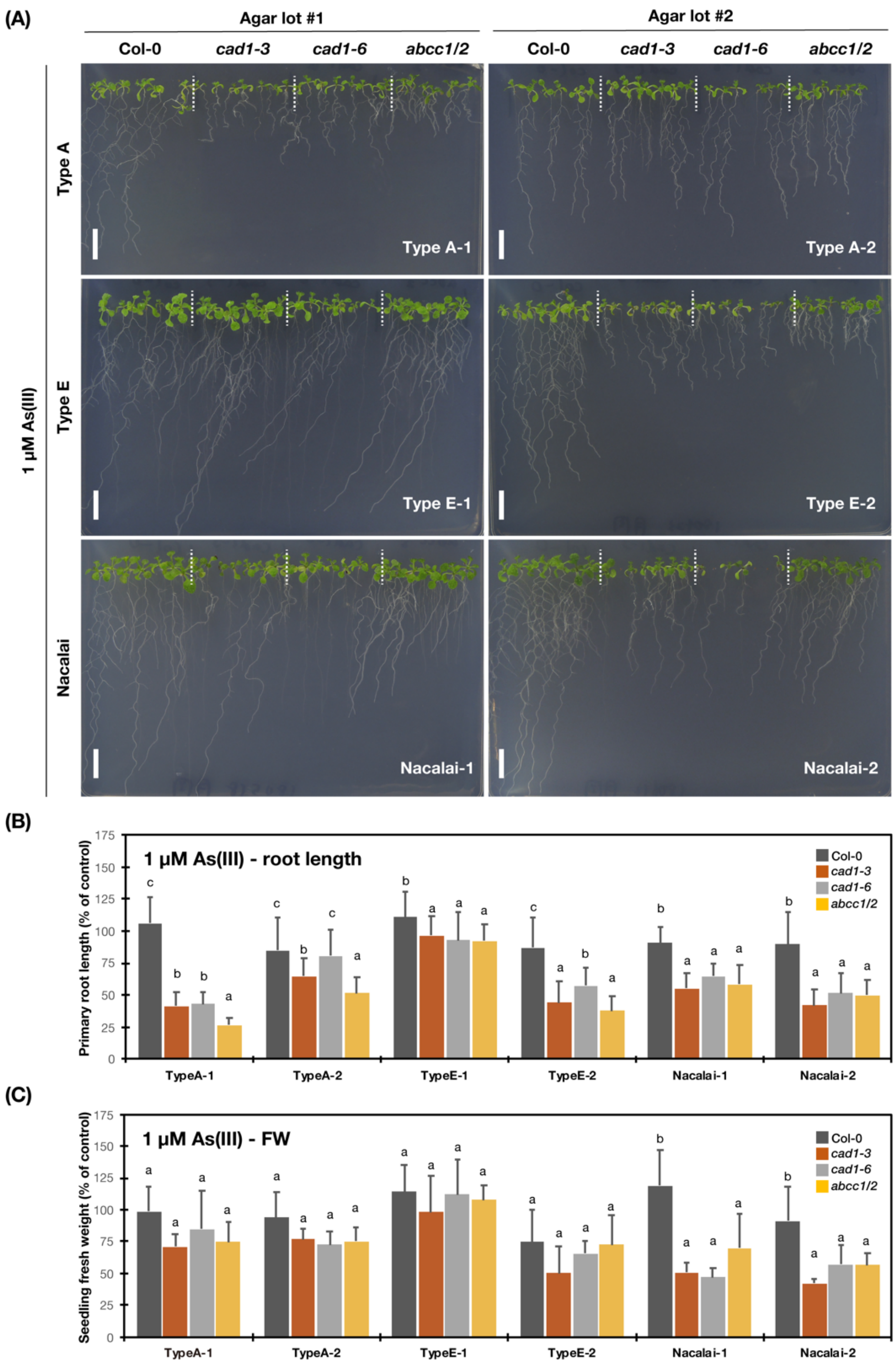

Supplementary Figure S5. As(III)-sensitivity of the phytochelatin-related Arabidopsis mutants on the agar plates prepared with different agar reagents. Three types of agar reagents with two independent lots for each type were tested. Col-0, *cad1-3*, *cad1-6* and *abcc1/2* were grown on the agar medium containing 1 µM As(III) for 12 d. **(A)** Phenotypes of Col-0, *cad1-3*, *cad1-6* and *abcc1/2*. Scale bars = 1 cm. **(B, C)** Relative primary root length **(B)** and fresh weight of seedlings **(C)** of Col-0, *cad1-3*, *cad1-6* and *abcc1/2*. Values are shown as percentage of each control. Data represent means with SD from at least three independent experiments (n = 23 – 28 for root length, and n = 3 – 4 for seedling fresh weight). Means sharing the same letter are not significantly different within each agar reagent ($P < 0.05$, Tukey's HSD).

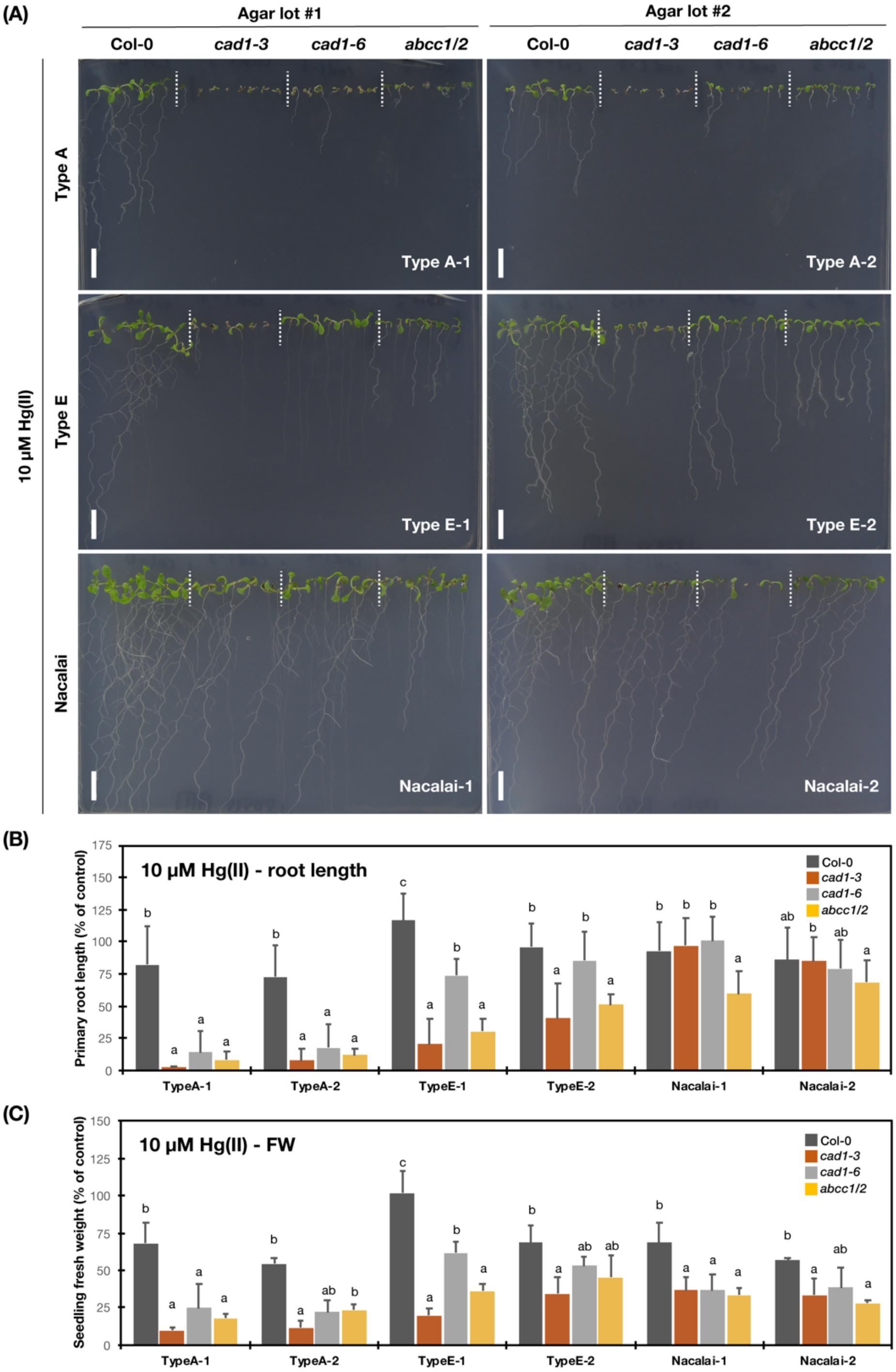

Supplementary Figure S6. Hg(II)-sensitivity of the phytochelatin-related Arabidopsis mutants on the agar plates prepared with different agar reagents. Three types of agar reagents with two independent lots for each type were tested. Col-0, *cad1-3*, *cad1-6* and *abcc1/2* were grown on the agar medium containing 10 µM Hg(II) for 12 d. **(A)** Phenotypes of Col-0, *cad1-3*, *cad1-6* and *abcc1/2*. Scale bars = 1 cm. **(B, C)** Relative primary root length **(B)** and fresh weight of seedlings **(C)** of Col-0, *cad1-3*, *cad1-6* and *abcc1/2*. Values are shown as percentage of each control. Data represent means with SD from at least three independent experiments (n = 14 – 28 for root length, and n = 3 – 4 for seedling fresh weight). Means sharing the same letter are not significantly different within each agar reagent ($P < 0.05$, Tukey's HSD).

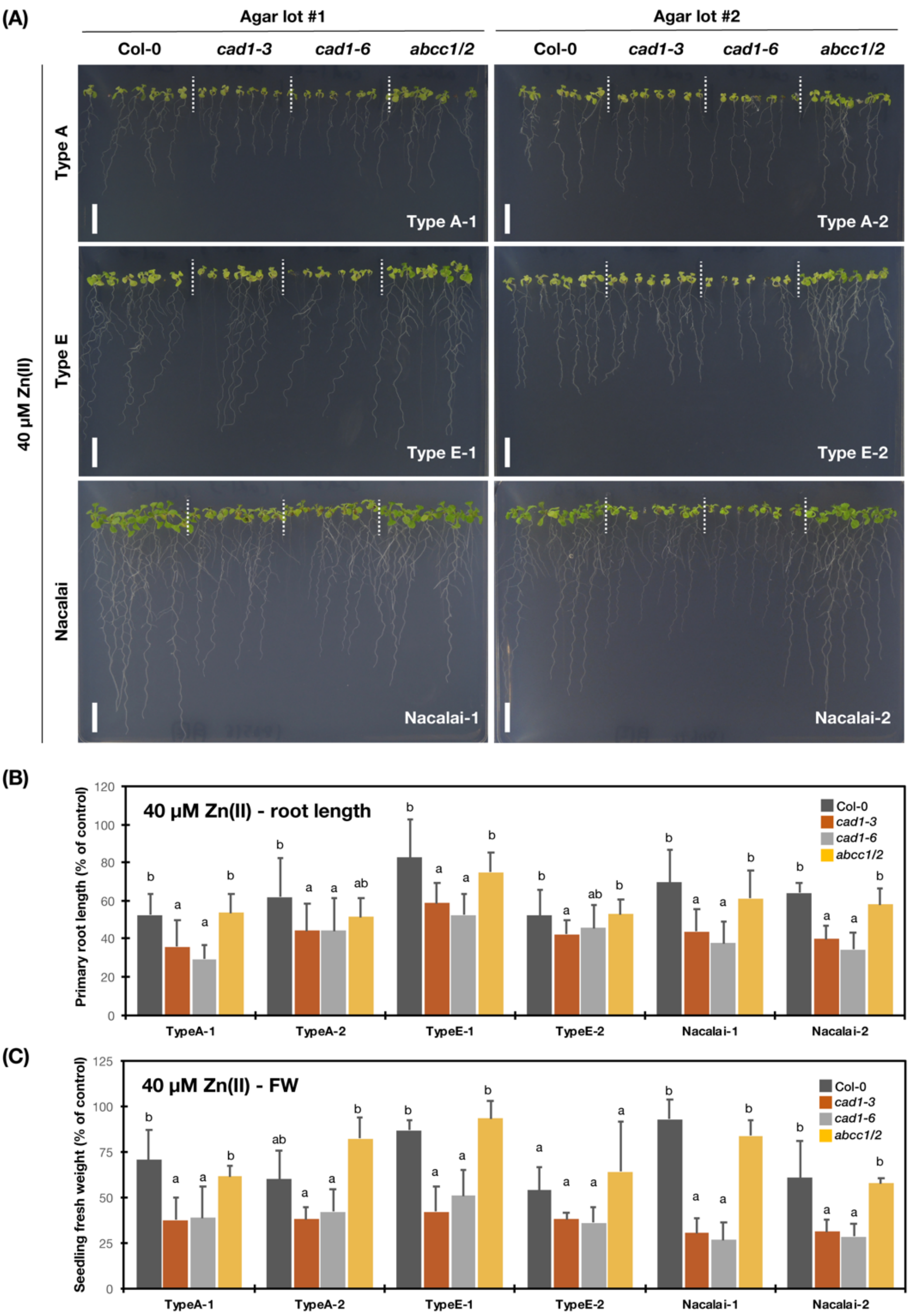

Supplementary Figure S7. Excess Zn(II)-sensitivity of the phytochelatin-related Arabidopsis mutants on the agar plates prepared with different agar reagents. Three types of agar reagents with two independent lots for each type were tested. Col-0, *cad1-3*, *cad1-6* and *abcc1/2* were grown on the agar medium containing 40 µM Zn(II) for 12 d. **(A)** Phenotypes of Col-0, *cad1-3*, *cad1-6* and *abcc1/2*. Scale bars = 1 cm. **(B, C)** Relative primary root length **(B)** and fresh weight of seedlings **(C)** of Col-0, *cad1-3*, *cad1-6* and *abcc1/2*. Values are shown as percentage of each control. Data represent means with SD from at least three independent experiments (n = 14 – 28 for root length, and n = 3 – 4 for seedling fresh weight). Means sharing the same letter are not significantly different within each agar reagent ($P < 0.05$, Tukey's HSD).

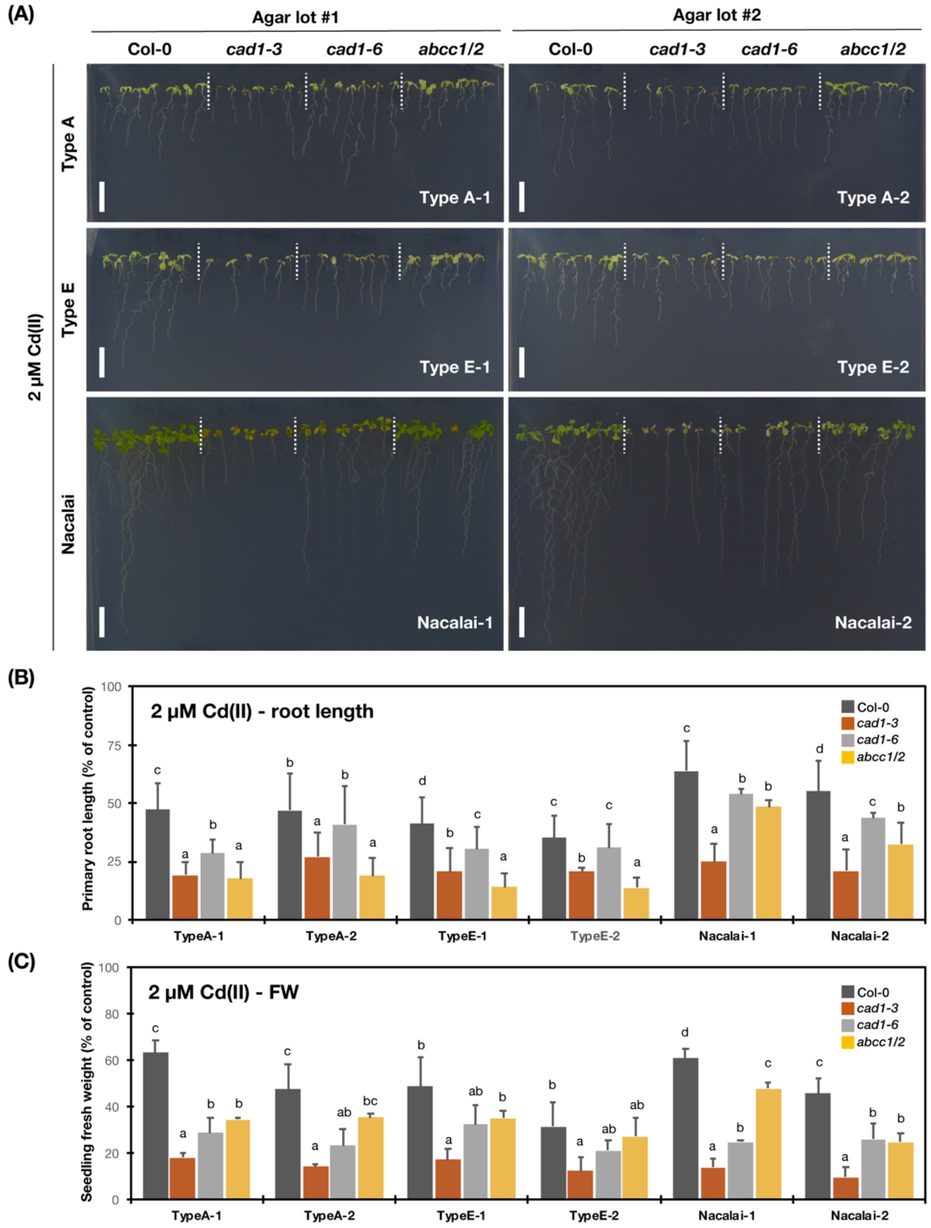

Supplementary Figure S8. Cd(II)-sensitivity of the phytochelatin-related Arabidopsis mutants on the agar plates prepared with different agar reagents. Three types of agar reagents with two independent lots for each type were tested. Col-0, *cad1-3*, *cad1-6* and *abcc1/2* were grown on the agar medium containing 2 µM Cd(II) for 12 d. **(A)** Phenotypes of Col-0, *cad1-3*, *cad1-6* and *abcc1/2*. Scale bars = 1 cm. **(B, C)** Relative primary root length **(B)** and fresh weight of seedlings **(C)** of Col-0, *cad1-3*, *cad1-6* and *abcc1/2*. Values are shown as percentage of each control. Data represent means with SD from at least three independent experiments (n = 23 – 28 for root length, and n = 3 – 4 for seedling fresh weight). Means sharing the same letter are not significantly different within each agar reagent ($P < 0.05$, Tukey's HSD).

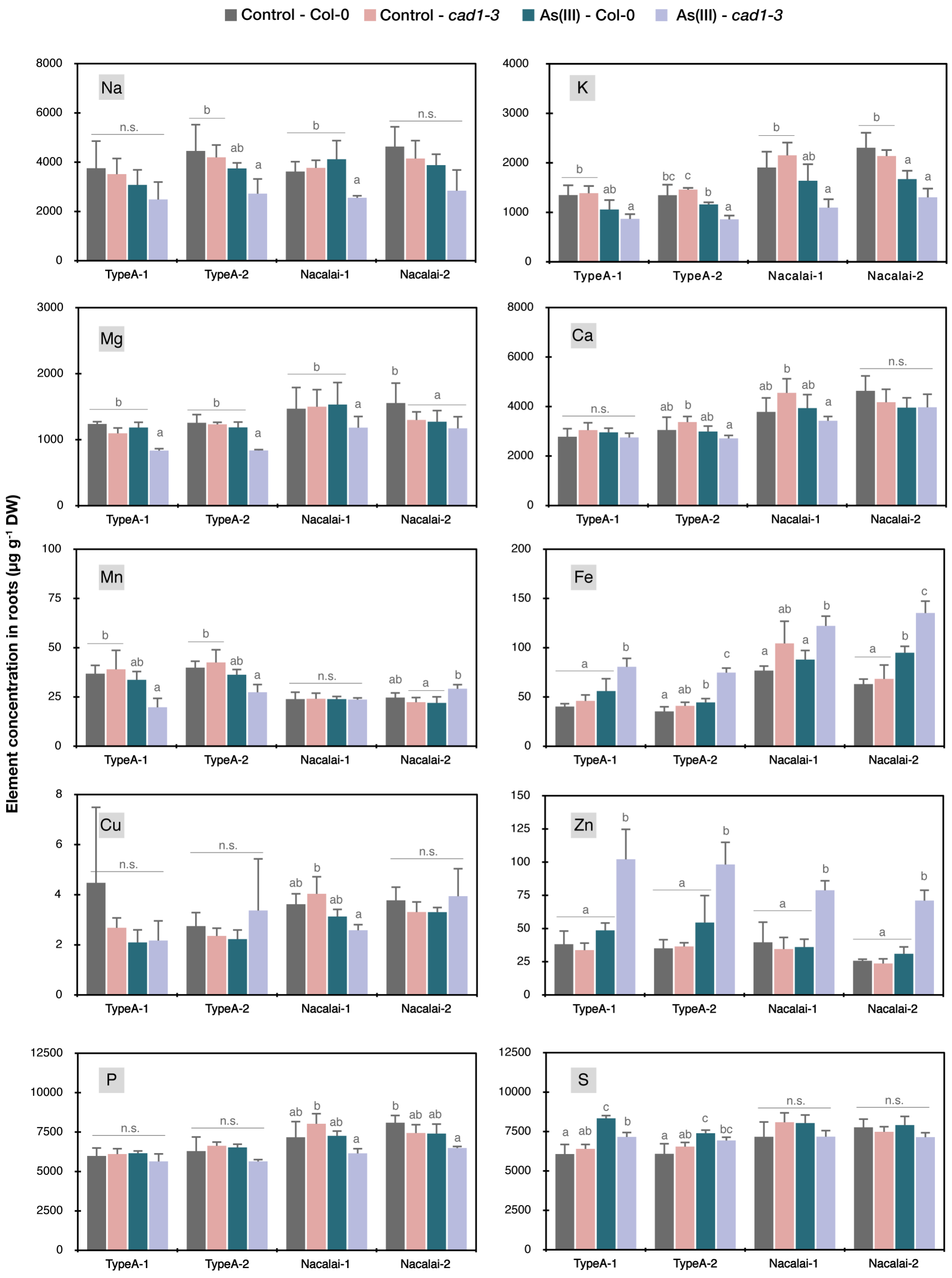

Supplementary Figure S9. Ionomic profiles in roots of Col-0 and *cad1-3* under normal conditions and As(III) stressed conditions. Plants were grown on the control medium prepared with the respective agar reagents for 10 d and then transferred to the fresh control medium or the medium containing 5 μM As(III). After 4 d, plants were harvested and concentrations of Na, K, Mg, Ca, Mn, Fe, Cu, Zn, P, and S in shoots were quantified by ICP-OES. Data represent means with SD from two independent experiments (n = 4). Means sharing the same letter are not significantly different within each agar reagent (*P* < 0.05, Tukey's HSD).

Table S1 Effects of As(III) treatment, genotypes and agar reagents on the ionomic profiles of Arabidopsis plants.

		Tretment[a]	Genotype[b]	Agar[c]	T x G[d]	T x A[e]	G x A[f]
Shoot	Na	***	***	***	***	***	***
	K	***	***	***	***	n.s.	n.s.
	Mg	***	***	***	***	n.s.	n.s.
	Ca	***	***	***	***	n.s.	n.s.
	Mn	***	***	***	***	*	*
	Fe	n.s.	*	***	n.s.	**	n.s.
	Cu	***	***	***	n.s.	***	n.s.
	Zn	***	***	***	***	n.s.	*
	P	***	*	***	n.s.	**	*
	S	***	***	***	***	***	n.s.
Root	Na	***	*	*	n.s.	n.s.	n.s.
	K	***	**	***	***	n.s.	n.s.
	Mg	***	***	***	**	n.s.	n.s.
	Ca	**	n.s.	***	*	n.s.	n.s.
	Mn	***	n.s.	***	n.s.	***	n.s.
	Fe	***	***	***	***	***	n.s.
	Cu	n.s.	n.s.	n.s.	n.s.	n.s.	n.s.
	Zn	***	***	***	***	*	n.s.
	P	***	*	***	***	n.s.	n.s.
	S	***	n.s.	***	***	***	n.s.

Elemental concentrations in shoots and roots of the Arabidopsis plants shown in Figure 7 and Supplementary Figure S6 were subjected to three-way ANOVA.

* $P < 0.05$, ** $P < 0.01$, *** $P < 0.001$, n.s. not significant.

[a] Effects of As(III) treatment

[b] Effects of genotypes (Col-0 and cad1-3)

[c] Effects of agar reagents supplemented to the media (TypeA-1, A-2, Nacalai-1 and Nacalai-2)

[d] Interaction effect between Treatment and Genotype

[e] Interaction effect between Treatment and Agar

[f] Interaction effect between Genotype and Agar
